# Differences in microbial community structure and metabolic activity among tea plantation soils under different management strategies

**DOI:** 10.3389/fmicb.2023.1219491

**Published:** 2023-08-02

**Authors:** Guoyou Li, Shaoxian Zhu, Jiang Long, Honglin Mao, Yonghong Dong, Yan Hou

**Affiliations:** ^1^College of Tea Science, Yunnan Agriculture University, Kunming, China; ^2^Xishuangbanna Luoboshanren Tea Co., Ltd., Menghai, China; ^3^Yunnan Pulis Biotechnology Co., Ltd., Kunming, China

**Keywords:** tea plant, management strategy, soil quality, soil microbial community, soil metabolite

## Abstract

**Introduction:**

Microorganisms play an important role in the multifunctionality of soil ecosystems. Soil microbial diversity and functions have a great impact on plant growth and development. The interactions between tea trees and soil microbiota can be linked with planting patterns and management strategies, whose effects on soil microbial community structure and metabolites are still unclear.

**Methods:**

Here we used amplicon sequencing and metabolomic analysis to investigate the differences in soil microbial composition and metabolites among three tea production systems: organic, non-organic, and intercropping.

**Results:**

We detected significant differences among the three systems and found that Firmicutes, Proteobacteria, Acidobacteriota, Actinobacteriota and Chloroflexi were the main bacteria in the three soil groups, although they varied in relative abundance. *Acidobacteria* bacterium increased significantly in the organic and intercropping groups. For fungi, Ascomycota and Basidiomycota were the main differential fungal phyla. Fungi alpha-diversity in the non-organic group was significantly higher than that in the other two groups, and was correlated with multiple soil physical and chemical factors. Moreover, network analysis showed that bacteria and fungi were strongly correlated. The changes in soil microorganisms caused by management and planting patterns may affect soil quality through corresponding changes in metabolites. Metabolomic analysis showed differences in metabolite composition among different groups. It was also found that the arachidonic acid metabolic pathway was affected by changes in soil microorganisms, and may further affect soil quality in an essential manner.

**Discussion:**

Planting patterns and management strategies may significantly affect soil microorganisms and therefore metabolites. Changes in soil microorganisms, especially in fungi, may alter soil quality by affecting soil physicochemical properties and metabolites. This study will provide new insights into soil quality monitoring from a microbiological perspective.

## 1. Introduction

Tea (*Camellia sinensis* L.), belonging to the family Theaceae, is an evergreen shrub or small tree whose leaves and leaf buds are used to produce tea (Kui et al., 2021b). Tea has become one of the most popular beverages in the world, with multiple health benefits (Trevisanato and Kim, 2000; Perez-Burillo et al., 2021; Bag et al., 2022). Tea tree is one of the most important economic crops in China. In 2019, tea planting area in China reached approximately 3.1 million hectares, with a total yield of 2.78 million tons (Xie et al., 2022). To maintain high yield and quality, chemical fertilizers, particularly nitrogen fertilizers, have been widely used. However, the long-term excessive application of fertilizers exerts negative impacts on soil and plants, leading to soil acidification, nutrient loss, and decreased tea quality (Yang et al., 2018; Wang et al., 2020). To address these problems, the application of organic fertilizer has become one of the most important agricultural practices in tea plantations these days (Huang et al., 2022; Ye et al., 2022).

Soils are a vast reservoir of biodiversity, containing myriad life forms that are essential to the functioning of ecosystems (Nielsen et al., 2015; Mishra et al., 2023). Rapid advances in high-throughput sequencing technology have deepened our understanding of the composition and functional roles of soil microorganisms. The soil microbial community governs the biogeochemical cycling pertaining to macronutrients, micronutrients, and other elements vital for the growth of plants and animals (Jansson and Hofmockel, 2020). It is influenced by and interacts with environmental factors, such as minerals, nutrients, redox conditions, and organic carbon composition, which may alter microbial diversity and richness (Jansson and Hofmockel, 2020). Changes in the composition and function of microbial communities can also influence the biogeochemical processes of carbon flow, further accelerating or mitigating climate change (Naylor et al., 2020). Studies have shown that any loss in microbial diversity will likely reduce the multifunctionality in terrestrial ecosystems, and damage ecosystem services such as nutrient cycling, soil fertility, primary production, and climate regulation (Delgado-Baquerizo et al., 2016; Kong et al., 2023; Wang et al., 2023).

In the past few years, attention has been diverted to the effects of plant-associated microbial community on plant growth and health (Pascale et al., 2019; Rai et al., 2023). The plant rhizosphere microbiome plays an important role in plant growth, yield, and disease resistance (Qu et al., 2020). Currently, various microbial taxa including beneficial bacteria and fungi, are used as biological fertilizers. They can improve plant nutrition by mobilizing or increasing the availability of nutrients in the soil, and thus have great potential to enhance soil fertility (Singh et al., 2008, 2010; Mitter et al., 2021). Microorganisms in the soil can improve soil fertility and provide nutrients for plants by decomposing litter as well (Hattenschwiler et al., 2005).

Applying exogenous organic matter helps to improve the balance and stability of soil microorganisms (Gryta et al., 2020). Changes in the levels of soil organic matter has the potential to alter bacterial microbiome, and thereby the macrophage activation of *Echinacea purpurea* root extracts (Haron et al., 2019). It was also found that using organic fertilizers can reinforce soil ability to suppress pathogenic fungi in the peanut rhizosphere (Chen et al., 2020). Overall, the application of organic fertilizer can promote microbial activities, enhance the synergistic effect within soil microbiome, increase the availability of soil organic matter and nutrients, and improve plant biomass (Zhang et al., 2019).

Tea planting systems depend highly on soil quality. The evaluation of soil quality under different management strategies and planting patterns is important for the production of organic tea. However, variations in soil microbial composition of different types of tea plantations and their due effects on soil quality are still unclear. In this study, we explored the microbial profiles and metabonomics of three soils of tea plantations: organic, non-organic, and intercropping to clarify the unique interactions between soil microbial community and metabolites, and their influences on soil properties, such as organic matter, total nitrogen, total phosphorus, and total potassium. This research will provide valuable insights into the improvement of soil quality in tea plantations through the use of microorganisms, and finally promoting tea plant growth.

## 2. Materials and methods

### 2.1. Soil sampling

Soil samples were collected from two tea plantations in Menghai County, Yunnan Province, southwestern China in August 2022. One plantation (latitude: 22°2′56″N, longitude: 100°37′48″E) was certified organic by Controllo e Certificazione Prodotti Biologici (CCPB, a renowned and professional inspection and certification body based in Italy for accrediting organic and eco-friendly production). *Docynia delavayi* trees (a wild fruit tree distributed in southwestern China) formed a natural intercropping system with tea trees in parts of the plantation. In the other plantation (latitude: 22°2′58″N, longitude: 100°37′44″E), non-organic practices were conducted, in which chemical fertilizers and pesticides were used. The two plantations were geographically close to each other. Three groups of soil samples were collected using a stainless steel spade from the following tea production systems of the two plantations: organic, non-organic, and intercropping. Soil of 10–20 cm deep and 5–15 cm near tea tree roots were taken. Each group included 10 samples. For each sample, five subsamples were collected in a zigzag pattern and mixed thoroughly. The well-mixed soil samples were carefully transferred to aseptic sampling bags and frozen at −80°C (Tedeschi and De Paoli, 2011) for further analysis.

### 2.2. Determination of soil characteristics

Soil pH was determined in a mixture of soil and water at a ratio of 1:5 (wt/vol) using pH strips (Zhang et al., 2019). Soil ammonia nitrogen (NH_4_) and nitrate nitrogen (NO_3_) were extracted with a 2 M KCL solution. Available potassium (AK) was determined by the atomic absorption method (Carter and Gregorich, 2007). Available phosphorus (AP) was determined based on the OD value at 880 nm by sodium bicarbonate extration, according to the Olsen method (Olsen, 1954). Total nitrogen (TN) was analyzed by fully burning each sample in a high-temperature reactor (Ma et al., 2017). Total phosphorus (TP) and total potassium (TK) were determined by NaOH molybdenum-antimony colorimetry method (Butkhup and Samappito, 2008). Organic matter (OM) was determined by a total organic matter analyzer (multi N/C 3100, Analytik Jena, Germany).

### 2.3. DNA extraction and sequencing

DNA was extracted from 0.5 g of soil using the Magnetic Soil and Stool DNA Kit (Tiangen, China) (Zhu et al., 2021). DNA concentrations were measured using a NanoDrop 2000-UV spectrophotometer (Thermo Scientific, Waltham, MA, United States). The 341 forward (5’-CCTAYGGGRBGCASCAG-3′) and 806 reverse (5′-GGACTACNNGGGTATCTAAT-3′) primers (Frank et al., 2013) were used to amplify the V3–4 region of the 16S rRNA gene, while the SSU0817 forward (5′-TTAGCATGGAATAATRRAATAGGA-3′) and 1,196 reverse (5′-TCTGGACCTGGTGAGTTTCC-3′) primers (Borneman and Hartin, 2000) were used to amplify the ITS1-F region of the 18S rRNA gene. PCR products were detected by 2% agarose gel electrophoresis. The target strip was recovered using a glue recovery kit (Qiagen, China). The library was sequenced using the Illumina NovaSeq sequencing platform. The raw sequencing data were uploaded to the public database National Center for Biotechnology Information (NCBI), with the accession number PRJNA983565.

### 2.4. Analysis of sequencing data

Raw tags were obtained by merging pair-ended reads using FLASH (V1.2.11, http://ccb.jhu.edu/software/FLASH/). Quality control was conducted on the raw tags using the fastp program to get high-quality clean tags, from which chimeras were detected and removed with Vsearch software (2.14.1) (Rognes et al., 2016). Then the DADA2 R package (Callahan et al., 2016) was used to denoise the sequences and generate amplicon sequence variants (ASVs) for further analysis. ASVs were later classified using the Naive Bayes classifier. Alpha-diversity values of the Shannon index and Chao1 index were calculated with the QIIME2 software (Bolyen et al., 2019). Bray–Curtis dissimilarity was calculated using the R-package vegan (v4.1.1) (Dixon, 2003) while PCoA analysis was performed using the ade4 R package (Dray and Dufour, 2007). LDA EFfect Size (LEfSe) (Segata et al., 2011) was conducted to identify differential markers between sample groups.

### 2.5. Data acquisition of metabolomic study based on liquid chromatography tandem mass spectrometry (LC-MS/MS)

One hundred mg of each soil sample was transferred to an Eppendorf tube and mixed with 1,000 μL of extraction solution (methanol: water = 3:1, isotope labeled internal standard). The mixture was homogenized at 35 Hz for 4 min and sonicated in an ice-water bath for 5 min (Alseekh et al., 2021). The homogenization and sonication cycle was repeated three times. The samples were incubated for 1 h at −40°C and centrifuged at 12000 rpm (RCF = 13,800 × g, *R* = 8.6 cm) for 15 min at 4°C (Alseekh et al., 2021). The obtained supernatant fluid was transferred to a fresh glass vial for analysis. Quality control (QC) samples were prepared by mixing an equal aliquot of the supernatants from all soil samples.

A Vanquish UHPLC system (Thermo Fisher Scientific, United States) was used for this study (Wang et al., 2016). The target compounds were separated by an Acquity^™^ UPLC HSS T3 column (100 mm × 2.1 mm, 1.8 μm). Eluent A was water containing 5 mmol/L ammonium acetate and 5 mmol/L acetic acid, while eluent B was acetonitrile. Column temperature was at 4°C and sample volume was 2 μL.

### 2.6. Soil metabolomic analysis

The original LC–MS/MS data were converted to mzXML format by ProteoWizard. XCMS was used for peak identification, peak extraction, peak alignment, and integration (Smith et al., 2006). Then BiotreeDB (V2.1) self-built secondary mass spectrum database was applied for material annotation. The cutoff value was set at 0.3. Deviations were filtered based on relative standard deviation (RSDS), namely coefficient of variation (CV). Only peak area data with no more than 50% null value in one group or no more than 50% hollow value in all groups were retained. Missing values in the original data were simulated. The numerical simulation method was used to fill in half of the minimum value. Then, the data were normalized to the internal standard peak intensity to generate a new data matrix. Partial least squares regression was used to establish the relationship model between metabolite expression and samples. Metabolites with a variable importance in projection (VIP) value >1 in OPLS-DA analysis and *p* < 0.05 in univariate analysis were considered significantly changed (Chong and Xia, 2018).

### 2.7. Statistical analysis

R software (v4.1.1) was used for statistical analysis. The Wilcoxon rank-sum test was used to compare differences in Shannon index and Chao1 index. PERMANOVA analysis was performed to assess differences in beta diversity between soil groups. Environmental indicators were statistically analyzed by *t*-test. Spearman correlation was used to investigate microbial metabolites and environmental factors. The significance threshold was set at |*r*| > 0.6 and *p* < 0.05. Network visualization and analysis were conducted using Gephi software (v0.9.2).

## 3. Results

### 3.1. Effects of different management strategies on soil physical and chemical properties

Differences in the main physical and chemical properties of soils under different management strategies were investigated. The results showed that the levels of TN, OM, AN (alkeline-N) and pH in intercropping group were increased significantly, followed by organic and non-organic groups. TK was significantly increased in organic group compared with non-organic group soil. Although AP and AK showed no significant difference among soil samples, the lowest values were found in the non-organic group (Table 1). These results revealed that soil characteristics may be affected by different management strategies and planting methods.

**Table 1:** Physicochemical properties of soils under different management systems.

	TN (g/kg)	TP (g/kg)	TK (g/kg)	AN (mg/kg)	AP (mg/kg)	AK (mg/kg)	OM (g/kg)	pH
Organic	1.020 ± 0.25^c^	0.95 ± 0.22	7.02 ± 0.069^b^	94.26 ± 13.45^c^	1.31 ± 0.026	172.97 ± 8.67	33.41 ± 4.29^c^	4.94 ± 0.059^c^
Non-organic	4.24 ± 0.13^b^	0.93 ± 0.032	10.85 ± 0.58^a^	240.19 ± 3.9^b^	1.66 ± 0.43	208.44 ± 51.18	110.461 ± 4.01^b^	5.26 ± 0.041^b^
Intercropping	6.35 ± 0.25^a^	1.16 ± 0.01	7.15 ± 0.098^b^	331.73 ± 8.67^a^	1.61 ± 0.31	204.18 ± 44.84	155.58 ± 0.90^a^	5.78 ± 0.149^a^

Data within a column without shared letters indicate significant differences at *p* < 0.05. Data represent the mean ± standard (*n* = 3 biological replicates). TN, total nitrogen; TP, total phosphorus; TK, total potassium; AN, alkeline-N; AK, available potassium; AP, available phosphorus; OM, organic matter.

### 3.2. Effects of different management strategies on soil microbial communities

**Figure 1:**
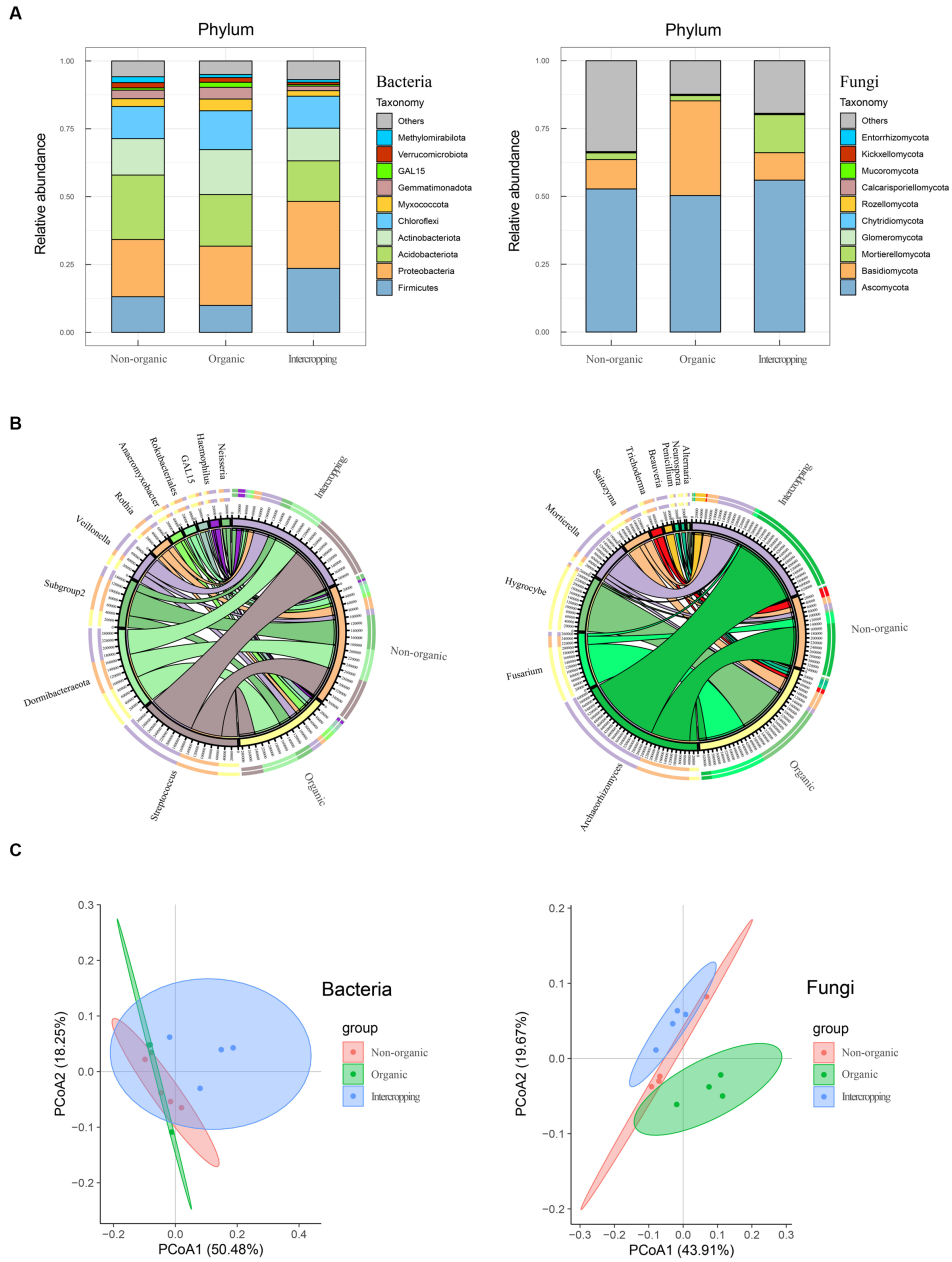
Composition analysis of soil microorganisms. Bacterial and fungal community compositions in organic, non-organic and intercropping groups at phylum **(A)** and genus **(B)** levels. Bacterial and fungal principal component analysis based on Bray–Curtis distance matrix **(C)**.

Considering the close relationship between soil characteristics and its microbial community, bacterial and fungal compositions of the three groups of soil were analyzed (four replicates for each sample group). A total of 761,015 and 928,360 high quality sequences were obtained in bacteria and fungi, respectively. The results of microbial annotation showed that Chloroflexi, Actinobacteriota, Acidobacteriota, Proteobacteria, and Firmicutes were the main phyla of bacteria, but their proportions vary among the three soil systems. The relative abundance of Acidobacteriota in non-organic group was higher than that in organic group, while Proteobacteria and Firmicutes were higher in intercropping group. The relative abundance of Firmicutes in organic group was lowest, while that of Actinobacteriota in organic group was highest (Figure 1A). For fungal composition at the phylum level, Ascomycota was dominant with the highest abundance in all three soil systems. Basidiomycota was mostly detected in organic group, while Mortierellomycota was mostly in intercropping group (Figure 1A). At the genus level, the microbial composition showed diversity among the three groups of samples. The top 10 genera of bacteria and fungi were analyzed (Figure 1B), among which *Streptococcus*, *AD3*, *Subgroup2*, *Veillonella*, and *Rothia* were the genera of bacteria that were abundant in soil. *Streptococcus* was most abundant in intercropping group, followed by non-organic and organic groups. *AD3* was dominant in organic group, while the abundance of *Subgroup2* was highest in non-organic group. *Hygrocybe* and *Fusarium* were the two fungi genera of highest relative abundance in organic group. In contrast, non-organic and intercropping groups were mainly dominated by *Archaeorhizomyces*, which had the highest abundance in intercropping group than in the other groups of soils. Besides, a certain abundance of *Mortierella* was detected in intercropping group.

**Figure 2:**
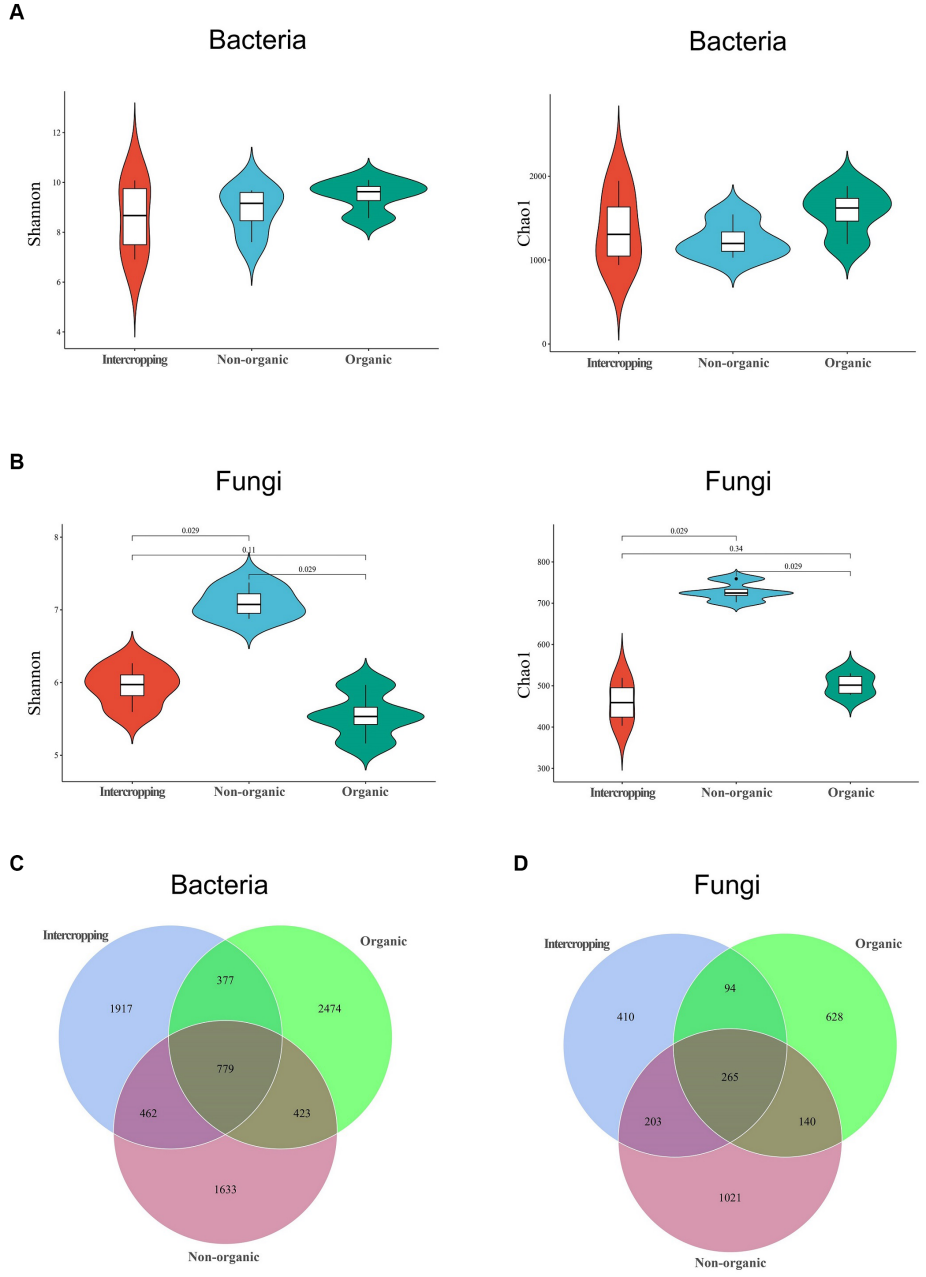
Microbial diversity in soils under different management systems. Diversity analysis of bacteria **(A)** and fungi **(B)** using the Shannon and Chao1 indices. Venn diagram analysis of bacterial **(C)** and fungal **(D)** species in the three soils.

To further explore the differences in bacterial and fungal community structure among different soil groups, principal coordinate analysis (PCoA) was performed (Figure 1C). We observed significant separation of fungal composition among the three types of soil, indicating that fungal community structure might be strongly affected by different strategies of soil management. By alpha-diversity analysis, we found no significant difference in bacterial diversity among the three groups (Figure 2A). However, significant differences were detected in fungal diversity, with non-organic group displaying the highest value (Figure 2B).

The numbers of shared and unique ASVs of bacteria and fungi of different soils are demonstrated in Venn diagrams. In terms of bacteria, 779 shared ASVs were detected among the three soils, with organic group having the most unique ASVs (2474) and non-organic group the least (1633) (Figure 2C). Regarding fungi, 265 shared ASVs were detected, with the largest number of unique ASVs in non-organic group (1021) and the smallest (410) in intercropping group (Figure 2D). The higher proportions of unique bacterial and fungal ASVs in each group revealed great differences among the three soils in microbial community structure.

### 3.3. Comparative analysis of microbial biomarkers of different soils

**Figure 3:**
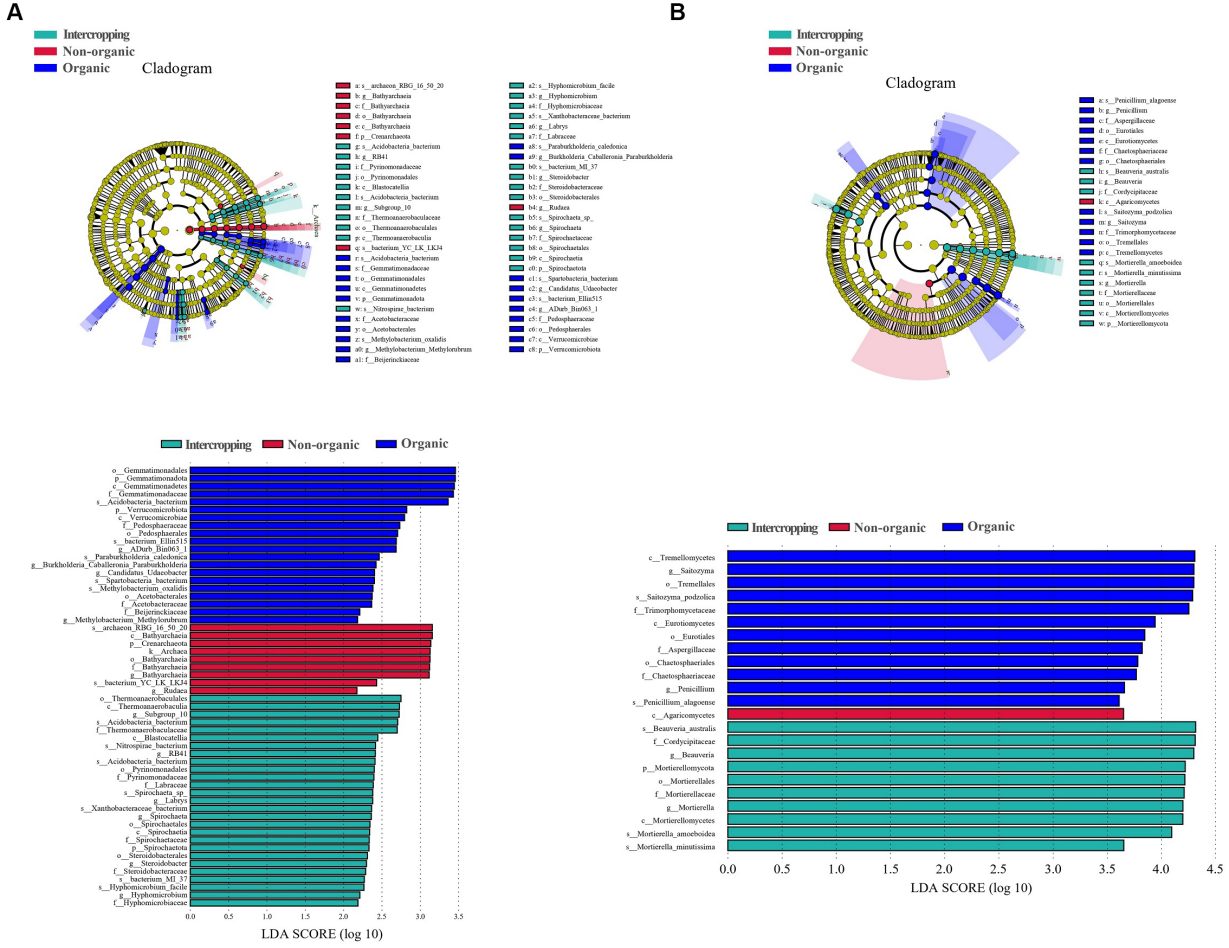
Differential microbiological analysis among different groups. LDA EFfect Size (LEfSe) analysis of soil bacteria groups **(A)**. LEfSe analysis of soil fungi groups **(B)**.

LEfSe analysis was used to identify microbial biomarkers, which showed significant differences in the species of bacteria (Figure 3A) and fungi (Figure 3B) among soils. In organic group, *Acidobacteria bacterium*, *bacterium Ellin515*, *Paraburkholderia caledonica*, *Spartobacteria bacterium*, and *Methylobacterium oxalidis* were the most abundant bacterial species. *Bathyarchaeia* and *Rudaea* were detected to be significantly enriched in non-organic group. *Steroidobacter*, *Nitrospirae bacterium*, *Acidobacteria bacterium*, *Spirochaeta* sp., *Xanthobacteraceae bacterium*, *bacterium MI-37*, *Hyphomicrobium facile* were significantly enriched in intercropping group. For fungal biomarkers, *Saitozyma podzolica* and *Penicillium alagoense* were significantly enriched in organic group, *Beauveria australis*, *Mortierella amoeboidea*, and *Mortierella minutissim* in intercropping group, while Agaricomycetes in non-organic group. In general, these microbial biomarkers may respond to planting patterns and management strategies to varying degrees, leading to the differences among soil samples.

### 3.4. Correlation analysis between fungi, bacteria, and environmental factors

To investigate fungal-bacterial interactions in tea plantation soils, the three groups of soils were mixed, and a correlation network analysis (|*r*| > 0.7, *p* < 0.05) was performed (Supplementary Figure S1A). Overall, the network consists of 146 nodes. Fungi involved 94 nodes (64.38%) while bacteria nodes accounted for only 35.62%, indicating that the network was dominated by fungal activities. The proportion of positive correlation was 59.24%, and that of negative correlation was 40.76%, revealing predominantly synergistic interactions within the bacterial-fungal community. The topological role of each ASV in the microbial network was demonstrated in a Zi-Pi plot to investigate the bacterial and fungal co-occurrence in tea plantation soils (Supplementary Figure S1B). We found that most ASVs were categorized as connectors, indicating a high degree of connectivity in symbiotic interactions between the bacterial and fungal communities. We thus assume that there may be strongly interacted species within the co-occurrence network, which may contribute to the stability of the network itself.

**Figure 4:**
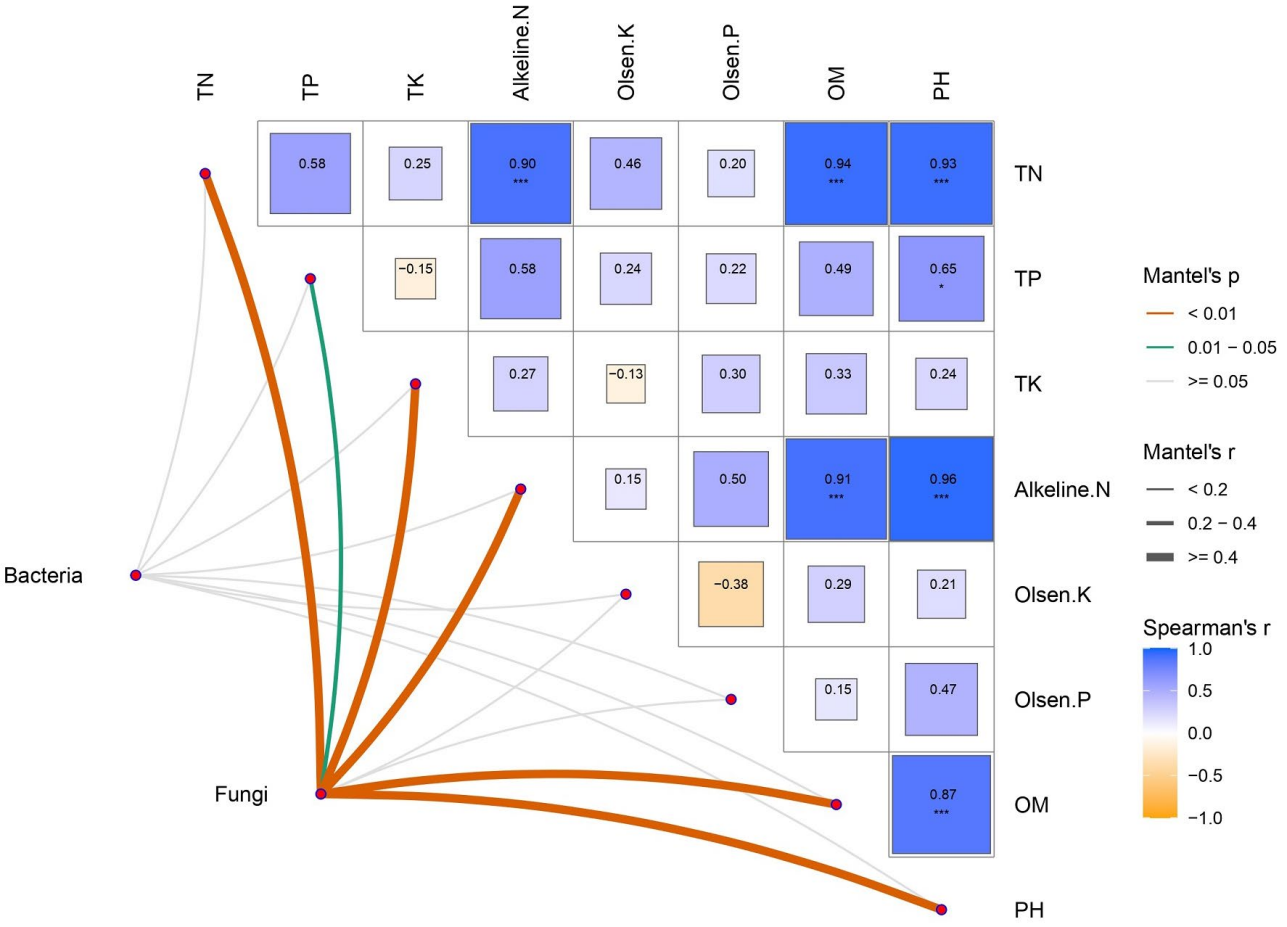
Correlations of soil microbial communities and physicochemical properties. Physicochemical properties are demonstrated in a heatmap constructed by Spearman correlation. Correlations between physicochemical properties and bacterial-fungal communities were determined using the Mantel test for correlation. Significance levels: ^*^p < 0.05, ^**^p < 0.01, ^***^p < 0.001.

Mantel test analysis was used to explore the relationship between soil microbial community and physical and chemical parameters. The results showed that soil physical and chemical properties were mostly positively correlated with each other, which had the most significant effect on the fungal community. Bacteria, however, responded poorly to soil physical and chemical changes (Figure 4).

### 3.5. Soil metabolite patterns and differential analysis

**Figure 5:**
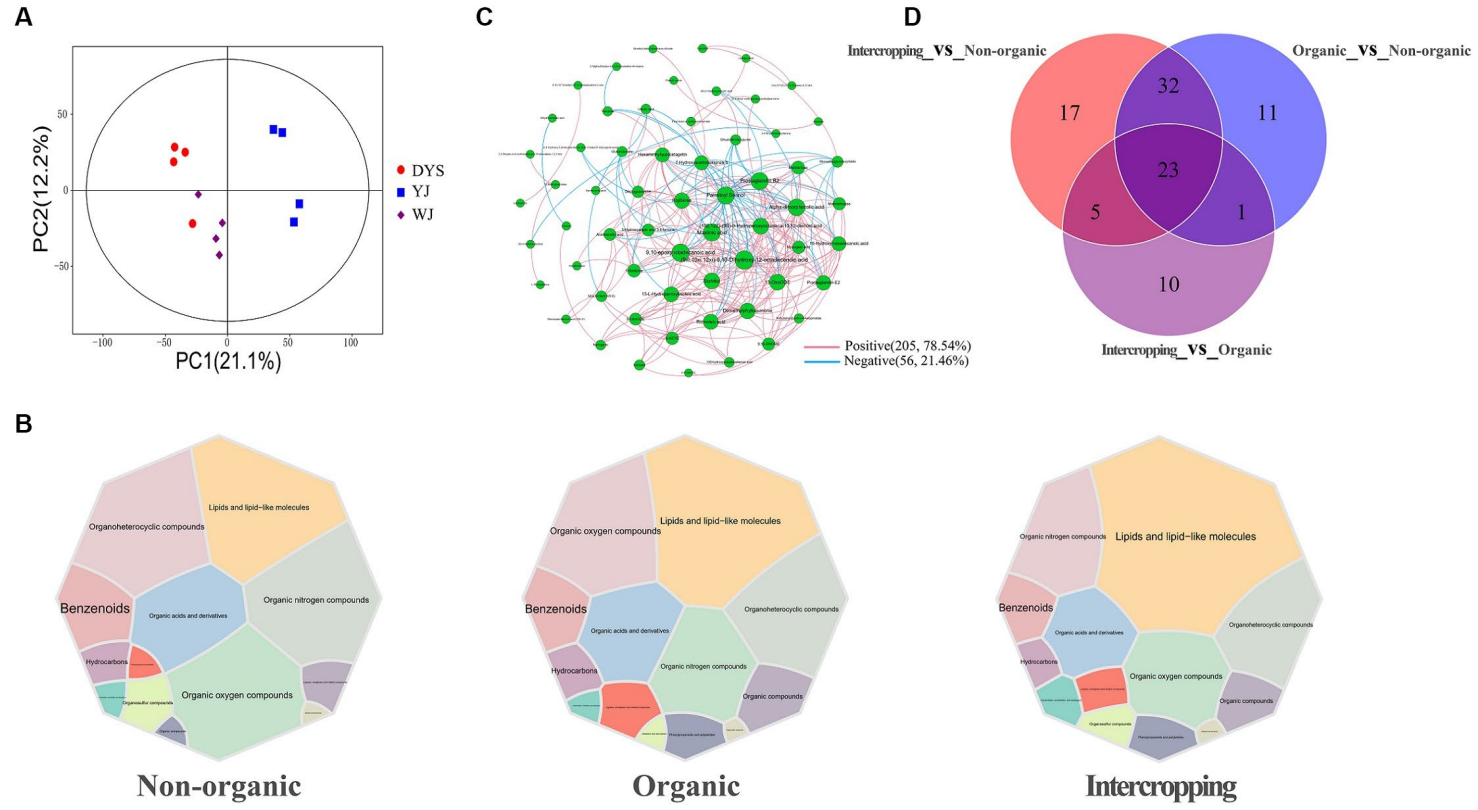
Analysis of metabolite patterns in different soils. **(A)** PCA analysis of soil metabolites in the three soils. **(B)** Main metabolites of the three groups of soils. **(C)** Correlation network analysis of soil metabolites. **(D)** Venn diagram analysis of soil differential metabolites.

Non-targeted metabolomic analysis was performed to unravel metabolic characteristics of different soils, and a total of 2,617 metabolites were identified. PCA was performed to establish the relationship between metabolite expression and soil samples (Figure 5A). An obvious separation was observed, indicating differences in the abundances of metabolites in soils managed under different systems.

Metabolites identified in the three soils overlapped extensively. The main metabolites included lipids and lipid-like molecules, organic nitrogen compounds, organoheterocyclic compounds, organic oxygen compounds, and organic acids and derivatives, although slight differences in metabolite abundances among the soils were observed (Figure 5B). A metabolite interaction network showed that the interaction patterns of metabolites were mostly positive (78.54%), with 9,10-epoxyoctadecanoic acid, (9xi,10xi,12xi)-9, 10-dihydroxy-12-octadecenoic acid, palmitoyl serinol, sorbitol, maslinic acid, and kojibiose showing high degrees of connectivity (Figure 5C). The highest number of differential metabolites were detected between intercropping and non-organic groups, while the lowest number between intercropping and organic groups. A total of 23 overlapping metabolites were found among the three groups of soil (Figure 5D). Most metabolites were increased in organic and intercropping groups, especially acetoacetic acid, kojibiose, and deoxyguanosine, which were significantly concentrated in the two soils (Supplementary Figures S2A,B). KEGG enrichment analysis was performed on differential metabolites between organic and non-organic groups, and intercropping and non-organic groups, respectively. It was found that the expression of ABC transporters was higher in organic and intercropping groups than in non-organic group. We also found significant differences in arachidonic acid metabolism, linoleic acid metabolism, and other metabolic pathways (Supplementary Figures S2C,D). Changes in these metabolic pathways may be one of the factors contributing to the differences in soil fertility under different management systems.

### 3.6. Regulatory network of soil differential metabolites, microorganisms, and environmental factors

**Figure 6:**
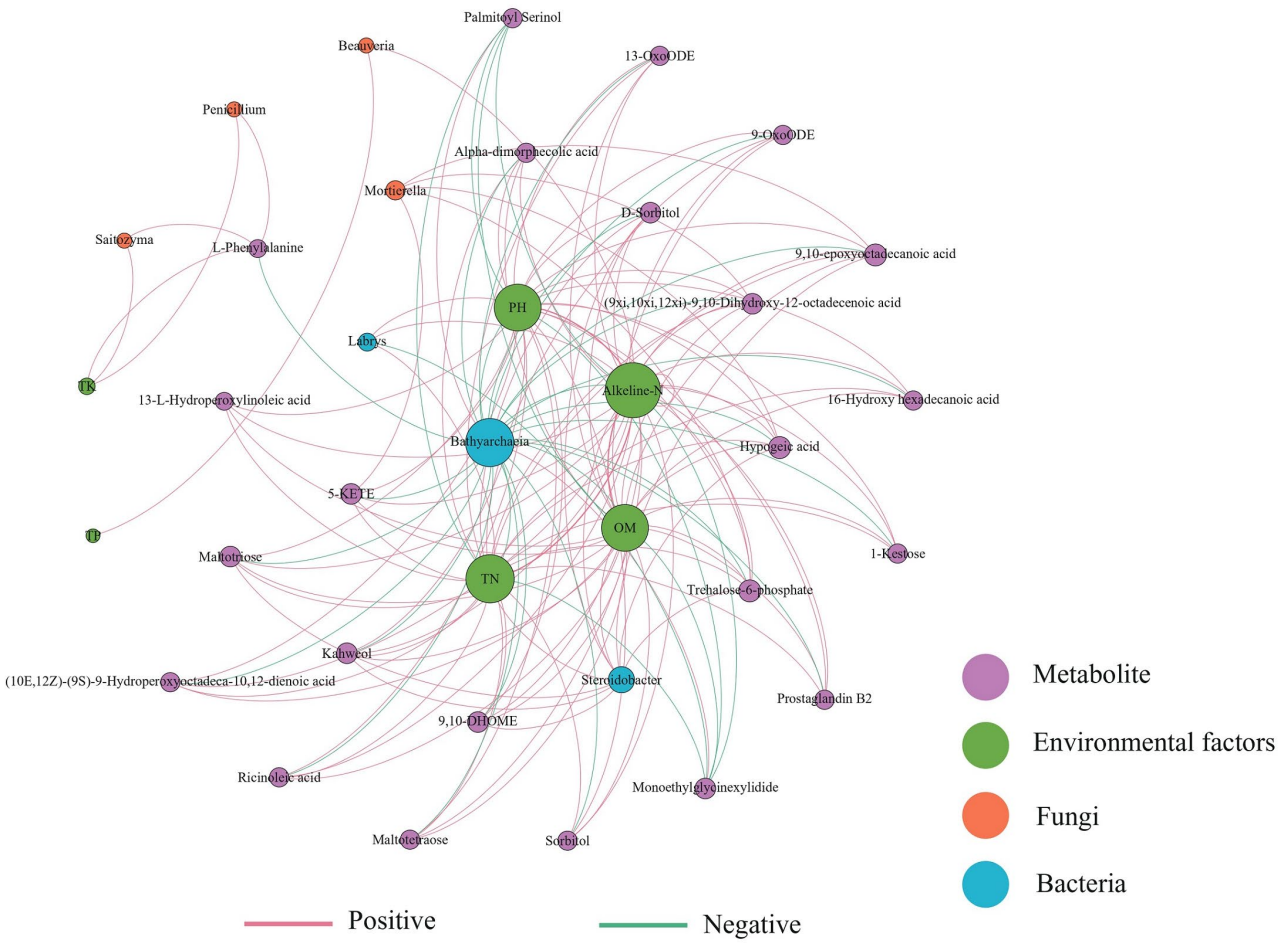
Correlation network of the soil physicochemical properties, soil differential metabolites, and microbial biomarkers (LDA score >2.0) (|Spearman’s *r*| >0.6, *p* < 0.05). The red line indicates a positive correlation and the blue line indicates a negative correlation.

A co-occurrence network was constructed based on bacterial-fungal communities, differential metabolites, and environmental factors of the three soils (Figure 6). *Bathyarchaeia* was negatively correlated with most metabolites and environmental factors, while *Steroidobacter* was positively correlated with metabolites. The fungus *Mortierella* was positively correlated with 9,10-epoxyoctadecanoic acid, hypogeic acid, 5-KETE, trehalose-6-phosphate, and other metabolites. The metabolite alkeline-N has high connectivity in the network and is strongly correlated with most factors. Soil physical and chemical properties such as pH, TN, and OM interact with most metabolites and microorganisms, and their changes may affect the composition of soil microorganisms and metabolites.

## 4. Discussion

Soil is one of the most important assets of planet earth, encompassing a large proportion of microscopic biodiversity, including prokaryotes and microscopic eukaryotes (Mishra et al., 2023). Most of the processes of nutrient availability and loss pathways in soil are mediated by microorganisms. In this study, we collected three groups of soil samples and explored their differences in physical and chemical properties, microbiome, and metabolite composition.

Tea cultivation intensity and duration have strong impacts on microbial community structure, microbial biomass and its functioning, likely through soil acidification and fertilizer addition (Han et al., 2007). Yan et al. (2020) found that the soil of tea plantations in China tended to become acidic, and the pH value of many sites dropped to less than 4.5, which was too acidic for tea growth, and may have adverse effects on soil microorganisms. In contrast, no significant soil acidification was observed in organic tea plantations. Data from several studies showed that fungi had a higher association with pH and were more susceptible to soil pH than bacteria. An increasing soil pH will significantly affect fungal community structure and total fungal biomass (Carrino-Kyker et al., 2016; Kui et al., 2021a). Fungal alpha and beta diversity had a greater effect on tea yield and quality than bacterial diversity (Tang et al., 2022). Plant growth may therefore be affected through changes in microbial community structure by altering soil pH.

It has been reported that soil microbial community structure and biological function can be improved by organic soil management, such as the use of organic fertilizers (Diacono and Montemurro, 2010). In this study, the highest bacterial diversity was detected in organic soil, although not statistically significant, which indirectly reflects that organic soil management may provide a more suitable environment for bacterial reproduction, resulting in a higher bacterial diversity and abundance than other management strategies. In contrast, the diversity of fungi in non-organic group was much higher than that in organic and intercropping groups, which may be related to soil pH. Chloroflexi, Actinobacteriota, Proteobacteria, and Firmicutes were the main bacterial phylum in all the three soils, while Basidiomycota, Ascomycota were the main phylum of fungi. This result is generally consistent with previous studies (Tan et al., 2019; Naumova et al., 2021; Kui et al., 2021a; Aira et al., 2022; Liu et al., 2022). Furthermore, we detected *Streptococcus* in soil microorganisms, and its relative abundance was highest in intercropping group but lowest in organic group. *Streptococcus* is a group of pathogenic bacteria mostly detected in the intestinal tract of humans and animals and are associated with a variety of diseases (Peng et al., 2020; Zhao et al., 2022). The genera *Streptococcus* detected in soil has been reported to be heavy metal resistant, and increase with the accumulation of heavy metals (Li et al., 2020). On the other hand, *Streptococcus* has the ability to degrade hydrocarbons and improve the quality of contaminated soil (Aqeel et al., 2021). Acidobacteria *Subgroup2* was significantly positively correlated with the production of phosphatase and may be involved in the degradation of organophosphorus (Mason et al., 2021). It had a higher relative abundance in non-organic group, which may be explained by a lower abundance of organophosphorus in this soil. We assume that the relative abundance of Acidobacteria *Subgroup2* was increased to compensate for the organophosphorus loss in non-organic managed soil. In terms of fungi, higher abundances of *Fusarium* and *Hygrocybe* were identified in organic soil. The *Fusarium* genus comprises important saprophytic and phytopathogenic fungi and is widespread in nature (Zubi et al., 2021). It spends most of its life cycle in soil and interacts extensively with soil microorganisms (Mukjang et al., 2022). A higher abundance of *Fusarium* in organic group might be caused by a high carbon level in the soil, which can shelter its conidia and thus supports its growth and survival (Logrieco et al., 1995; Zubi et al., 2021). *Hygrocybe* is believed to be related to C and N cycles (Carron et al., 2020). Organic soil containing more *Hygrocybe* may be beneficial for soil carbon utilization. The relative abundance of *Archaeorhizomyces* in intercropping group was much higher than that in non-organic and organic groups. Previous studies have found that the relative abundance of *Archaeorhizomyces* in soil is positively correlated with the application of bioferfertilizer and may promote plant growth (Zhang et al., 2018). *Acidobacteria bacterium* are abundant in soil and are an important component of the soil microbial community (Kalam et al., 2020). We found that they were significantly enriched in organic and intercropping groups compared with non-organic group. Genomes of *Acidobacteria bacterium* encode a wide range of carbohydrate-active enzymes, which are involved in the decomposition, utilization, and biosynthesis of various carbohydrates (Dedysh and Sinninghe Damsté, 2018). Studies have found that the significant difference in the distribution of *Acidobacteria bacterium* among soils is mainly caused by the input of N and pH values (Liu et al., 2017). Therefore, we speculated that the enrichment of this bacterial species in organic group might be related to the high level of carbon and organic matter in the soil (Dedysh and Sinninghe Damsté, 2018). We also found that *Bathyarchaeia* was significantly enriched in non-organic soil and may be negatively correlated with a variety of soil metabolites and environmental factors. *Bathyarchaeia* is closely related to soil pH, EC, and levels of Na^+^and Cl^−^ in salt-stressed soil (Wang et al., 2019). This suggests that *Bathyarchaeia* may play specific roles in regulating ecological functions in different soil environments. Some studies suggested that the improvement of soil fertility by organic fertilizer and soil regulator might decrease the relative abundance of the soil bacterium *Steroidobacter* (Wang et al., 2021). *Steroidobacter* was significantly detected in intercropping group in this study and was negatively correlated with a variety of metabolites. *Steroidobacter* may affect soil quality through the interactions with soil metabolites.

Metabolites in soil are mainly produced by plant roots and soil microorganisms (Kalu et al., 2021). A number of soil bacteria produce both volatile and soluble compounds, which likely play important roles in long-distance microbial interactions (Tyc et al., 2017). Study has found that Kojibios has a promoting effect on the growth of potential probiotic strains of *Bifidobacterium*, *Lactobacillus*, and *Streptococcus* (Garcia-Cayuela et al., 2014). It has been detected in soybean root exudates (Timotiwu and Sakurai, 2002), but its effect on plant soil remains unclear. Here we found that Kojibiose is the major differential metabolite in organic group and may be essential in the overall soil metabolic network. *Mortierella* has been reported to survive under unfavorable environmental conditions, promote plant growth, reduce chemical fertilizers and pesticides, and enhance crop yield (Ozimek and Hanaka, 2021). We found that *Mortierella* is positively correlated with the abundances of 9,10-epoxy octadecanoic acid, hypogeic acid, 5-KETE, trehalose-6-phosphate, and other metabolites. Trehalose metabolism in rhizobia is key for signaling plant growth, yield, and adaptation to abiotic stress, and its manipulation has a major agronomical impact on leguminous plants (Suarez et al., 2008). *Mortierella* has also been suggested to produce arachidonic acid (Botha et al., 1999). Organic acids and fatty acids were potential metabolites mediating the plant-bacteria interaction in the tea rhizosphere (Sun et al., 2022). The metabolic pathways of arachidonic acid and linolenic acid were detected to be different among soils. Studies have found that arachidonic acid is the main allelopathic substance affecting the interactions between the fungus Arbuscular mycorrhizal and bacteria (Lu et al., 2023). At the same time, arachidonic acid can also recruit beneficial microorganisms to the host rhizosphere to promote plant growth and soil nutrient turnover (Lu et al., 2023). Differences in the metabolic pathway of arachidonic acid among soils may be caused by varying microbial abundances, such as *Mortierella*, which may affect the growth and development of tea trees.

## 5. Conclusion

By exploring microbial and metabolite composition in soils of tea plantations under different management strategies, we detected significant differences in bacterial and fungal community compositions between organic, non-organic, and intercropping groups. Changes in soil pH might affect the composition of microorganisms, especially fungi. Soil metabolites are rich in lipids and lipid-like molecules, organic nitrogen compounds, and organoheterocyclic compounds, most of which are positively correlated. Changes in soil microbial community also affected the metabolic pathway of arachidonic acid, which is an important compound that influences soil quality. Importantly, we assume that soil quality of tea plantations may be influenced by varying microbial compositions through different metabolic pathways and their metabolites in the soil. This study will provide a basis for the improvement of soil fertility from the perspective of soil microorganisms by investigating the effects of microbial changes on soil quality and clarifying the underlying mechanisms.

## Data availability statement

The datasets presented in this study can be found in online repositories. The names of the repository/repositories and accession number(s) can be found at: https://www.ncbi.nlm.nih.gov/, PRJNA983565.

## Author contributions

GL and SZ wrote the manuscript. YH designed the experiments and revised the manuscript. JL and HM collected soil samples and assisted in interpreting results, and provided insights for writing the manuscript. GL, SZ, and YD analyzed the data and completed visualization. All authors contributed to the article and approved the submitted version.

## Funding

This research was funded by Special Project of Basic Research in Yunnan Province (202301AS070083); National Key Research and Development Program of China (2022YFD1200505); and 2021 “Three Regions” Scientific and Technological Talents Reward Program of Yunnan Province (A3032021156051).

## Conflict of interest

JL was employed by Xishuangbanna Luoboshanren Tea Co., Ltd.

YD was employed by Yunnan Pulis Technology Co., Ltd.

The remaining authors declare that the research was conducted in the absence of any commercial or financial relationships that could be construed as a potential conflict of interest.

## Publisher’s note

All claims expressed in this article are solely those of the authors and do not necessarily represent those of their affiliated organizations, or those of the publisher, the editors and the reviewers. Any product that may be evaluated in this article, or claim that may be made by its manufacturer, is not guaranteed or endorsed by the publisher.

## References

[ref1] Aira M. Perez-Losada M. Crandall K. A. Dominguez J. (2022). Composition, structure and diversity of soil bacterial communities before, during and after transit through the gut of the earthworm *Aporrectodea caliginosa*. Microorganisms 10:1025. doi: 10.3390/microorganisms10051025, PMID: 35630467 PMC9144582

[ref2] Alseekh S. Aharoni A. Brotman Y. Contrepois K. D’auria J. Ewald J.. (2021). Mass spectrometry-based metabolomics: a guide for annotation, quantification and best reporting practices. Nat. Methods 18, 747–756. doi: 10.1038/s41592-021-01197-1 34239102 PMC8592384

[ref3] Aqeel A. Hussain Z. Aqeel Q.-U.-A. Zafar J. Ehsan N. Tariq M. (2021). Enrichment and characterization of hydrocarbon degrading bacteria from various oil-contaminated sites in Pakistan. Geomicrobiol J. 38, 577–587. doi: 10.1080/01490451.2021.1903625

[ref4] Bag S. Mondal A. Majumder A. Banik A. (2022). Tea and its phytochemicals: hidden health benefits & modulation of signaling cascade by phytochemicals. Food Chem. 371:131098. doi: 10.1016/j.foodchem.2021.131098, PMID: 34634647

[ref5] Bolyen E. Rideout J. R. Dillon M. R. Bokulich N. Abnet C. C. Al-Ghalith G. A.. (2019). Reproducible, interactive, scalable and extensible microbiome data science using QIIME 2. Nat. Biotechnol. 37, 852–857. doi: 10.1038/s41587-019-0209-9, PMID: 31341288 PMC7015180

[ref6] Borneman J. Hartin R. J. (2000). Pcr primers that amplify fungal rRna genes from environmental samples. Appl. Environ. Microbiol. 66, 4356–4360. doi: 10.1128/AEM.66.10.4356-4360.2000, PMID: 11010882 PMC92308

[ref7] Botha A. Paul I. Roux C. Kock J. L. F. Coetzee D. J. Strauss T.. (1999). An isolation procedure for arachidonic acid producing *Mortierella* species. Anton. Leeuw. Int. J. Gen. Mol. Microbiol. 75, 253–256. doi: 10.1023/A:1001848709005 10427414

[ref8] Butkhup L. Samappito S. (2008). An analysis on flavonoids contents in Mao Luang fruits of fifteen cultivars (*Antidesma bunius*), grown in Northeast Thailand. Pak. J. Biol. Sci. 11, 996–1002. doi: 10.3923/pjbs.2008.996.1002, PMID: 18810968

[ref9] Callahan B. J. Mcmurdie P. J. Rosen M. J. Han A. W. Johnson A. J. A. Holmes S. P. (2016). DADA2: high-resolution sample inference from Illumina amplicon data. Nat. Methods 13:581. doi: 10.1038/nmeth.3869, PMID: 27214047 PMC4927377

[ref10] Carrino-Kyker S. R. Kluber L. A. Petersen S. M. Coyle K. P. Hewins C. R. Deforest J. L.. (2016). Mycorrhizal fungal communities respond to experimental elevation of soil Ph and P availability in temperate hardwood forests. FEMS Microbiol. Ecol. 92:fiw024. doi: 10.1093/femsec/fiw024, PMID: 26850158

[ref11] Carron A. I. Garibaldi L. A. Marquez S. Fontenla S. (2020). The soil fungal community of native woodland in Andean Patagonian Forest: a case study considering experimental forest management and seasonal effects. For. Ecol. Manag. 461:117955. doi: 10.1016/j.foreco.2020.117955

[ref12] Carter M. R. Gregorich E. G. (2007). Soil sampling and methods of analysis, Boca Raton, FL, CRC Press.

[ref13] Chen D. Wang X. Zhang W. Zhou Z. Ding C. Liao Y.. (2020). Persistent organic fertilization reinforces soil-borne disease suppressiveness of rhizosphere bacterial community. Plant Soil 452, 313–328. doi: 10.1007/s11104-020-04576-3

[ref14] Chong J. Xia J. (2018). MetaboAnalystR: an R package for flexible and reproducible analysis of metabolomics data. Bioinformatics 34, 4313–4314. doi: 10.1093/bioinformatics/bty528, PMID: 29955821 PMC6289126

[ref15] Dedysh S. N. Sinninghe Damsté J. S. (2018). Acidobacteria. Encycl. Life Sci. doi: 10.1002/9780470015902.a0027685

[ref16] Delgado-Baquerizo M. Maestre F. T. Reich P. B. Jeffries T. C. Gaitan J. J. Encinar D.. (2016). Microbial diversity drives multifunctionality in terrestrial ecosystems. Nat. Commun. 7:10541. doi: 10.1038/ncomms10541, PMID: 26817514 PMC4738359

[ref17] Diacono M. Montemurro F. (2010). Long-term effects of organic amendments on soil fertility. A review. Agron. Sustain. Dev. 30, 401–422. doi: 10.1051/agro/2009040

[ref18] Dixon P. (2003). Vegan, a package of R functions for community ecology. J. Veg. Sci. 14, 927–930. doi: 10.1111/j.1654-1103.2003.tb02228.x

[ref19] Dray S. Dufour A.-B. (2007). The ade4 package: implementing the duality diagram for ecologists. J. Stat. Softw. 22, 1–20. doi: 10.18637/jss.v022.i04

[ref20] Frank K. L. Rogers D. R. Olins H. C. Vidoudez C. Girguis P. R. (2013). Characterizing the distribution and rates of microbial sulfate reduction at Middle Valley hydrothermal vents. ISME J. 7, 1391–1401. doi: 10.1038/ismej.2013.17, PMID: 23535916 PMC3695286

[ref21] Garcia-Cayuela T. Diez-Municio M. Herrero M. Carmen Martinez-Cuesta M. Pelaez C. Requena T.. (2014). Selective fermentation of potential prebiotic lactose-derived oligosaccharides by probiotic bacteria. Int. Dairy J. 38, 11–15. doi: 10.1016/j.idairyj.2014.03.012

[ref22] Gryta A. Frac M. Oszust K. (2020). Genetic and metabolic diversity of soil microbiome in response to exogenous organic matter amendments. Agronomy 10:546. doi: 10.3390/agronomy10040546

[ref23] Han W. Kemmitt S. J. Brookes P. C. (2007). Soil microbial biomass and activity in Chinese tea gardens of varying stand age and productivity. Soil Biol. Biochem. 39, 1468–1478. doi: 10.1016/j.soilbio.2006.12.029

[ref24] Haron M. H. Tyler H. L. Chandra S. Moraes R. M. Jackson C. R. Pugh N. D.. (2019). Plant microbiome-dependent immune enhancing action of *Echinacea purpurea* is enhanced by soil organic matter content. Sci. Rep. 9:136. doi: 10.1038/s41598-018-36907-x, PMID: 30644442 PMC6333828

[ref25] Hattenschwiler S. Tiunov A. V. Scheu S. (2005). Biodiversity and litter decomposition interrestrial ecosystems. Annu. Rev. Ecol. Evol. Syst. 36, 191–218. doi: 10.1146/annurev.ecolsys.36.112904.151932

[ref26] Huang D. Wang Y. Chen X. Wu J. Wang H. Tan R.. (2022). Application of tea-specific fertilizer combined with organic fertilizer improves aroma of green tea. Horticulturae 8:950. doi: 10.3390/horticulturae8100950

[ref27] Jansson J. K. Hofmockel K. S. (2020). Soil microbiomes and climate change. Nat. Rev. Microbiol. 18, 35–46. doi: 10.1038/s41579-019-0265-7 31586158

[ref28] Kalam S. Basu A. Ahmad I. Sayyed R. Z. El-Enshasy H. A. Dailin D. J.. (2020). Recent understanding of soil *Acidobacteria* and their ecological significance: a critical review. Front. Microbiol. 11:580024. doi: 10.3389/fmicb.2020.580024, PMID: 33193209 PMC7661733

[ref29] Kalu C. M. Ogola H. J. O. Selvarajan R. Tekere M. Ntushelo K. (2021). Correlations between root metabolomics and bacterial community structures in the *Phragmites australis* under acid mine drainage-polluted wetland ecosystem. Curr. Microbiol. 79:34. doi: 10.1007/s00284-021-02748-7 34962589 PMC8714630

[ref30] Kong Z. Zhong H. Jin X. Cai Q. Zou Z. Ge G.. (2023). Linking microbial community structure to function underneath Moss-dominated biocrusts in rare earth elements mine areas under a subtropical climate. Land Degrad. Dev. doi: 10.1002/ldr.4737

[ref31] Kui L. Xiang G. Wang Y. Wang Z. Li G. Li D.. (2021a). Large-scale characterization of the soil microbiome in ancient tea plantations using high-throughput 16s Rrna and internal transcribed spacer amplicon sequencing. Front. Microbiol. 12:745225. doi: 10.3389/fmicb.2021.745225 34721345 PMC8555698

[ref32] Kui L. Xiang G. Wang Y. Wang Z. Li G. Li D.. (2021b). Large-scale characterization of the soil microbiome in ancient tea plantations using high-throughput 16s rRna and internal transcribed spacer amplicon sequencing. Front. Microbiol. 12:745225. doi: 10.3389/fmicb.2021.745225, PMID: 34721345 PMC8555698

[ref33] Li Q. You P. Hu Q. Leng B. Wang J. Chen J.. (2020). Effects of co-contamination of heavy metals and total petroleum hydrocarbons on soil bacterial community and function network reconstitution. Ecotoxicol. Environ. Saf. 204:111083. doi: 10.1016/j.ecoenv.2020.111083, PMID: 32791359

[ref34] Liu C. Dong Y. Hou L. Deng N. Jiao R. (2017). *Acidobacteria* community responses to nitrogen dose and form in Chinese fir plantations in southern China. Curr. Microbiol. 74, 396–403. doi: 10.1007/s00284-016-1192-8, PMID: 28184989

[ref35] Liu S. Wu J. Wang H. Lukianova A. Tokmakova A. Jin Z.. (2022). Soil layers impact *Lithocarpus* soil microbial composition in the Ailao Mountains subtropical Forest, Yunnan, China. J. Fungi 8:948. doi: 10.3390/jof8090948 PMC9504396 36135673

[ref36] Logrieco A. Moretti A. Ritieni A. Bottalico A. Corda P. (1995). Occurrence and toxigenicity of *Fusarium proliferatum* from preharvest maize ear rot, and associated mycotoxins, in Italy. Plant Dis. 79, 727–731. doi: 10.1094/PD-79-0727

[ref37] Lu P. Shi H. Tao J. Jin J. Wang S. Zheng Q.. (2023). Metagenomic insights into the changes in the rhizosphere microbial community caused by the root-knot nematode *Meloidogyne incognita* in tobacco. Environ. Res. 216:114848. doi: 10.1016/j.envres.2022.114848, PMID: 36403441

[ref38] Ma Y.-H. Fu S.-L. Zhang X.-P. Zhao K. Chen H. Y. H. (2017). Intercropping improves soil nutrient availability, soil enzyme activity and tea quantity and quality. Appl. Soil Ecol. 119, 171–178. doi: 10.1016/j.apsoil.2017.06.028

[ref39] Mason L. M. Eagar A. Patel P. Blackwood C. B. Deforest J. L. (2021). Potential microbial bioindicators of phosphorus mining in a temperate deciduous forest. J. Appl. Microbiol. 130, 109–122. doi: 10.1111/jam.14761, PMID: 32619072

[ref40] Mishra A. Singh L. Singh D. (2023). Unboxing the black box—one step forward to understand the soil microbiome: a systematic review. Microb. Ecol. 85, 669–683. doi: 10.1007/s00248-022-01962-5, PMID: 35112151 PMC9957845

[ref41] Mitter E. K. Tosi M. Obregón D. Dunfield K. E. Germida J. J. (2021). Rethinking crop nutrition in times of modern microbiology: innovative biofertilizer technologies. Front. Sustain. Food Syst. 5. doi: 10.3389/fsufs.2021.606815

[ref42] Mukjang N. Mombrikotb S. B. Bell T. (2022). Microbial community succession in steam-sterilized greenhouses infected with *Fusarium oxysporum*. Environ. Microbiol. Rep. 14, 577–583. doi: 10.1111/1758-2229.13072, PMID: 35445561 PMC9544407

[ref43] Naumova N. Belanov I. Alikina T. Kabilov M. (2021). Soil microbiome after nine years of fly ash dump spontaneous revegetation. Soil Res. 59, 673–683. doi: 10.1071/SR20304

[ref44] Naylor D. Sadler N. Bhattacharjee A. Graham E. B. Anderton C. R. Mcclure R.. (2020). Soil microbiomes under climate change and implications for carbon cycling. Annu. Rev. Environ. Resour. 45, 29–59. doi: 10.1146/annurev-environ-012320-082720

[ref45] Nielsen U. N. Wall D. H. Six J. (2015). Soil biodiversity and the environment. Annu. Rev. Environ. Resour. 40, 63–90. doi: 10.1146/annurev-environ-102014-021257

[ref46] Olsen S. R. (1954). Estimation of available phosphorus in soils by extraction with sodium bicarbonate (No. 939). US Department of Agriculture.

[ref47] Ozimek E. Hanaka A. (2021). *Mortierella* species as the plant growth-promoting fungi present in the agricultural soils. Agriculture 11:7. doi: 10.3390/agriculture11010007

[ref48] Pascale A. Proietti S. Pantelides I. S. Stringlis I. A. (2019). Modulation of the root microbiome by plant molecules: the basis for targeted disease suppression and plant growth promotion. Front. Plant Sci. 10:1741. doi: 10.3389/fpls.2019.01741 32038698 PMC6992662

[ref49] Peng Z. Cheng S. Kou Y. Wang Z. Jin R. Hu H.. (2020). The gut microbiome is associated with clinical response to anti-PD-1/PD-L1 immunotherapy in gastrointestinal cancer. Cancer Immunol. Res. 8, 1251–1261. doi: 10.1158/2326-6066.CIR-19-1014, PMID: 32855157

[ref50] Perez-Burillo S. Navajas-Porras B. Lopez-Maldonado A. Hinojosa-Nogueira D. Pastoriza S. Rufian-Henares J. A. (2021). Green tea and its relation to human gut microbiome. Molecules 26:3907. doi: 10.3390/molecules26133907, PMID: 34206736 PMC8271705

[ref51] Qu Q. Zhang Z. Peijnenburg W. J. G. M. Liu W. Lu T. Hu B.. (2020). Rhizosphere microbiome assembly and its impact on plant growth. J. Agric. Food Chem. 68, 5024–5038. doi: 10.1021/acs.jafc.0c00073 32255613

[ref52] Rai S. Omar A. F. Rehan M. Al-Turki A. Sagar A. Ilyas N.. (2023). Crop microbiome: their role and advances in molecular and Omic techniques for the sustenance of agriculture. Planta 257:27. doi: 10.1007/s00425-022-04052-5 36583789

[ref53] Rognes T. Flouri T. Nichols B. Quince C. Mahe F. (2016). Vsearch: a versatile open source tool for metagenomics. PeerJ 4:e2584. doi: 10.7717/peerj.2584, PMID: 27781170 PMC5075697

[ref54] Segata N. Izard J. Waldron L. Gevers D. Miropolsky L. Garrett W. S.. (2011). Metagenomic biomarker discovery and explanation. Genome Biol. 12:R60. doi: 10.1186/gb-2011-12-6-r60, PMID: 21702898 PMC3218848

[ref55] Singh S. Pandey A. Kumar B. Palni L. M. S. (2010). Enhancement in growth and quality parameters of tea [*Camellia sinensis* (L.) O. Kuntze] through inoculation with arbuscular mycorrhizal fungi in an acid soil. Biol. Fertil. Soils 46, 427–433. doi: 10.1007/s00374-010-0448-x

[ref56] Singh S. Pandey A. Palni L. M. S. (2008). Screening of arbuscular mycorrhizal fungal consortia developed from the rhizospheres of natural and cultivated tea plants for growth promotion in tea [*Camellia sinensis* (L.) O. Kuntze]. Pedobiologia 52, 119–125. doi: 10.1016/j.pedobi.2008.06.001

[ref57] Smith C. A. Want E. J. O'maille G. Abagyan R. Siuzdak G. (2006). XCMS: processing mass spectrometry data for metabolite profiling using nonlinear peak alignment, matching, and identification. Anal. Chem. 78, 779–787. doi: 10.1021/ac051437y, PMID: 16448051

[ref58] Suarez R. Wong A. Ramirez M. Barraza A. Orozco M. D. C. Cevallos M. A.. (2008). Improvement of drought tolerance and grain yield in common bean by overexpressing trehalose-6-phosphate synthase in rhizobia. Mol. Plant-Microbe Interact. 21, 958–966. doi: 10.1094/MPMI-21-7-0958, PMID: 18533836

[ref59] Sun L. Wang Y. Ma D. Wang L. Zhang X. Ding Y.. (2022). Differential responses of the rhizosphere microbiome structure and soil metabolites in tea (*Camellia sinensis*) upon application of cow manure. BMC Microbiol. 22:55. doi: 10.1186/s12866-022-02470-9, PMID: 35164712 PMC8842532

[ref60] Tan L. Gu S. Li S. Ren Z. Deng Y. Liu Z.. (2019). Responses of microbial communities and interaction networks to different management practices in tea plantation soils. Sustainability 11:4428. doi: 10.3390/su11164428

[ref61] Tang S. Zhou J. Pan W. Tang R. Ma Q. Xu M.. (2022). Impact of N application rate on tea (*Camellia sinensis*) growth and soil bacterial and Fungi communities. Plant Soil 475, 343–359. doi: 10.1007/s11104-022-05372-x

[ref62] Tedeschi R. De Paoli P. (2011). Collection and preservation of frozen microorganisms. Methods Mol. Biol. 675, 313–326. doi: 10.1007/978-1-59745-423-0_18 20949399

[ref63] Timotiwu P. B. Sakurai N. (2002). Identification of mono-, oligo-, and polysaccharides secreted from soybean roots. J. Plant Res. 115, 77–85. doi: 10.1007/s102650200012, PMID: 12884130

[ref64] Trevisanato S. I. Kim Y. I. (2000). Tea and health. Nutr. Rev. 58, 1–10. doi: 10.1111/j.1753-4887.2000.tb01818.x, PMID: 10697388

[ref65] Tyc O. Song C. Dickschat J. S. Vos M. Garbeva P. (2017). The ecological role of volatile and soluble secondary metabolites produced by soil bacteria. Trends Microbiol. 25, 280–292. doi: 10.1016/j.tim.2016.12.002, PMID: 28038926

[ref66] Wang M. Chen S. Chen L. Wang D. (2019). Saline stress modifies the effect of cadmium toxicity on soil archaeal communities. Ecotoxicol. Environ. Saf. 182:109431. doi: 10.1016/j.ecoenv.2019.109431, PMID: 31301593

[ref67] Wang T. Duan Y. Lei X. Cao Y. Liu L. Shang X.. (2023). Tea plantation intercropping legume improves soil ecosystem multifunctionality and tea quality by regulating rare bacterial taxa. Agronomy 13:1110:1110. doi: 10.3390/agronomy13041110

[ref68] Wang Z. Geng Y. Liang T. (2020). Optimization of reduced chemical fertilizer use in tea gardens based on the assessment of related environmental and economic benefits. Sci. Total Environ. 713:136439. doi: 10.1016/j.scitotenv.2019.136439, PMID: 31954250

[ref69] Wang R. Hou T. Sun Q. Ji L. Lei J. Zhang J. (2021). Organic fertilizers and soil conditioner recover chemical fertilizer-induced changes in soil bacterial community diversity in wine grape rhizosphere soil. Pol. J. Environ. Stud. 30, 1853–1863. doi: 10.15244/pjoes/126236

[ref70] Wang J. Zhang T. Shen X. Liu J. Zhao D. Sun Y.. (2016). Serum metabolomics for early diagnosis of esophageal squamous cell carcinoma by UHPLC-QTOF/MS. Metabolomics 12:116. doi: 10.1007/s11306-016-1050-5

[ref71] Xie S. Yang F. Feng H. Yu Z. Wei X. Liu C.. (2022). Potential to reduce chemical fertilizer application in tea plantations at various spatial scales. Int. J. Environ. Res. Public Health 19:5243. doi: 10.3390/ijerph19095243, PMID: 35564638 PMC9103282

[ref72] Yan P. Wu L. Wang D. Fu J. Shen C. Li X.. (2020). Soil acidification in Chinese tea plantations. Sci. Total Environ. 715:136963. doi: 10.1016/j.scitotenv.2020.136963, PMID: 32014781

[ref73] Yang X.-D. Ni K. Shi Y.-Z. Yi X.-Y. Zhang Q.-F. Fang L.. (2018). Effects of long-term nitrogen application on soil acidification and solution chemistry of a tea plantation in China. Agric. Ecosyst. Environ. 252, 74–82. doi: 10.1016/j.agee.2017.10.004

[ref74] Ye J. Wang Y. Wang Y. Hong L. Jia X. Kang J.. (2022). Improvement of soil acidification in tea plantations by long-term use of organic fertilizers and its effect on tea yield and quality. Front. Plant Sci. 13:1055900. doi: 10.3389/fpls.2022.1055900, PMID: 36618668 PMC9822707

[ref75] Zhang J. Bei S. Li B. Zhang J. Christie P. Li X. (2019). Organic fertilizer, but not heavy liming, enhances banana biomass, increases soil organic carbon and modifies soil microbiota. Appl. Soil Ecol. 136, 67–79. doi: 10.1016/j.apsoil.2018.12.017

[ref76] Zhang F. Huo Y. Cobb A. B. Luo G. Zhou J. Yang G.. (2018). Trichoderma biofertilizer links to altered soil chemistry, altered microbial communities, and improved grassland biomass. Front. Microbiol. 9:848. doi: 10.3389/fmicb.2018.00848, PMID: 29760689 PMC5937142

[ref77] Zhao X. Jiang L. Fang X. Guo Z. Wang X. Shi B.. (2022). Host-microbiota interaction-mediated resistance to inflammatory bowel disease in pigs. Microbiome 10:115. doi: 10.1186/s40168-022-01303-1, PMID: 35907917 PMC9338544

[ref78] Zhu N. Zhu Y. Kan Z. Li B. Cao Y. Jin H. (2021). Effects of two-stage microbial inoculation on organic carbon turnover and fungal community succession during co-composting of cattle manure and rice straw. Bioresour. Technol. 341:125842. doi: 10.1016/j.biortech.2021.125842, PMID: 34469819

[ref79] Zubi W. S. M. Mohd M. H. Nor N. M. I. M. Zakaria L. (2021). *Fusarium* species in mangrove soil in northern peninsular Malaysia and the soil physico-chemical properties. Microorganisms 9:497. doi: 10.3390/microorganisms9030497 33652900 PMC7996719

