# Supplementary figures and images for: Differences in microbial community structure and metabolic activity among tea plantation soils under different management strategies
Source: Front Microbiol. 2023 Aug 2;14:1219491. doi: 10.3389/fmicb.2023.1219491 (PMC10433390; doi:10.3389/fmicb.2023.1219491)

A

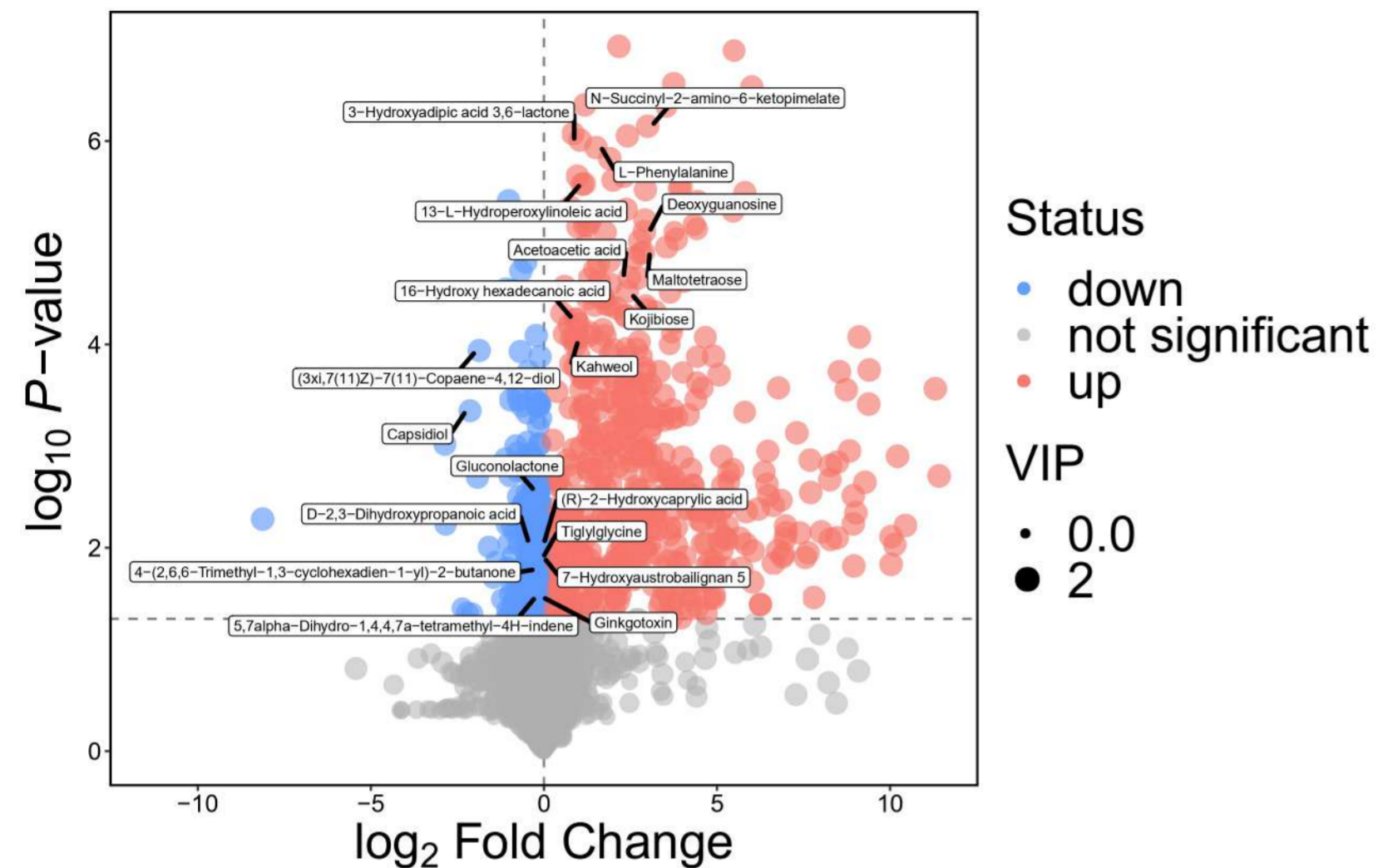

B

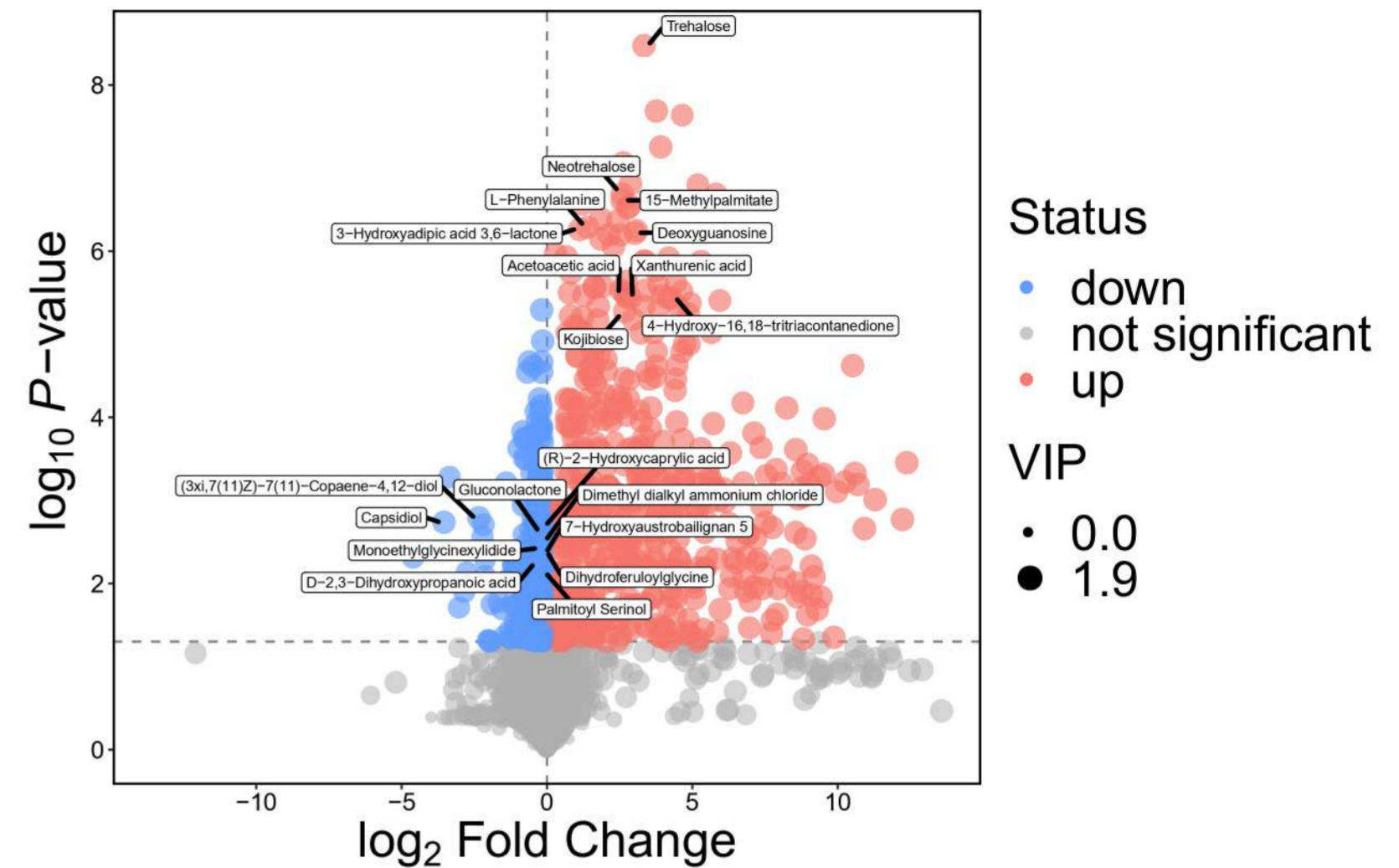

C

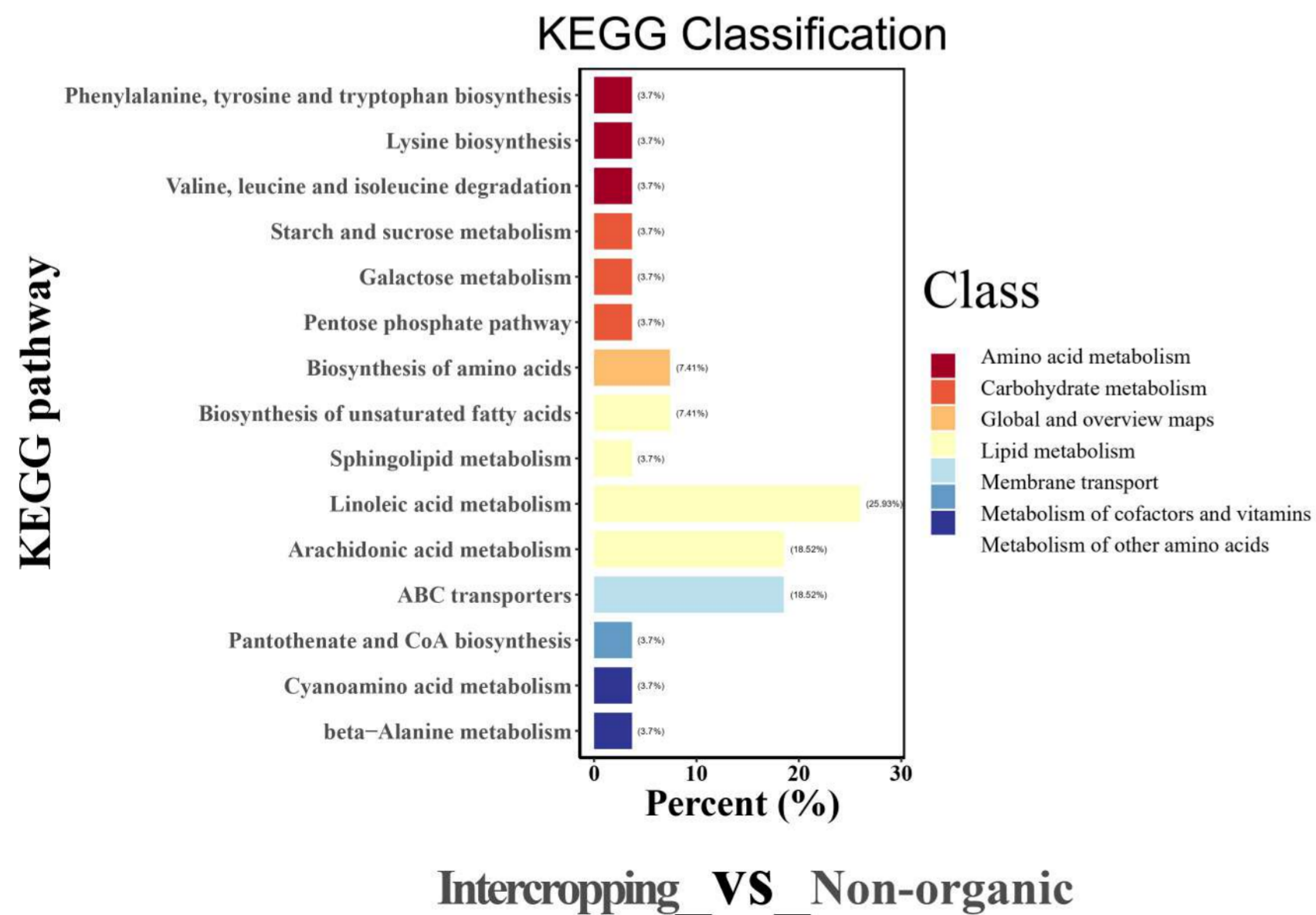

D

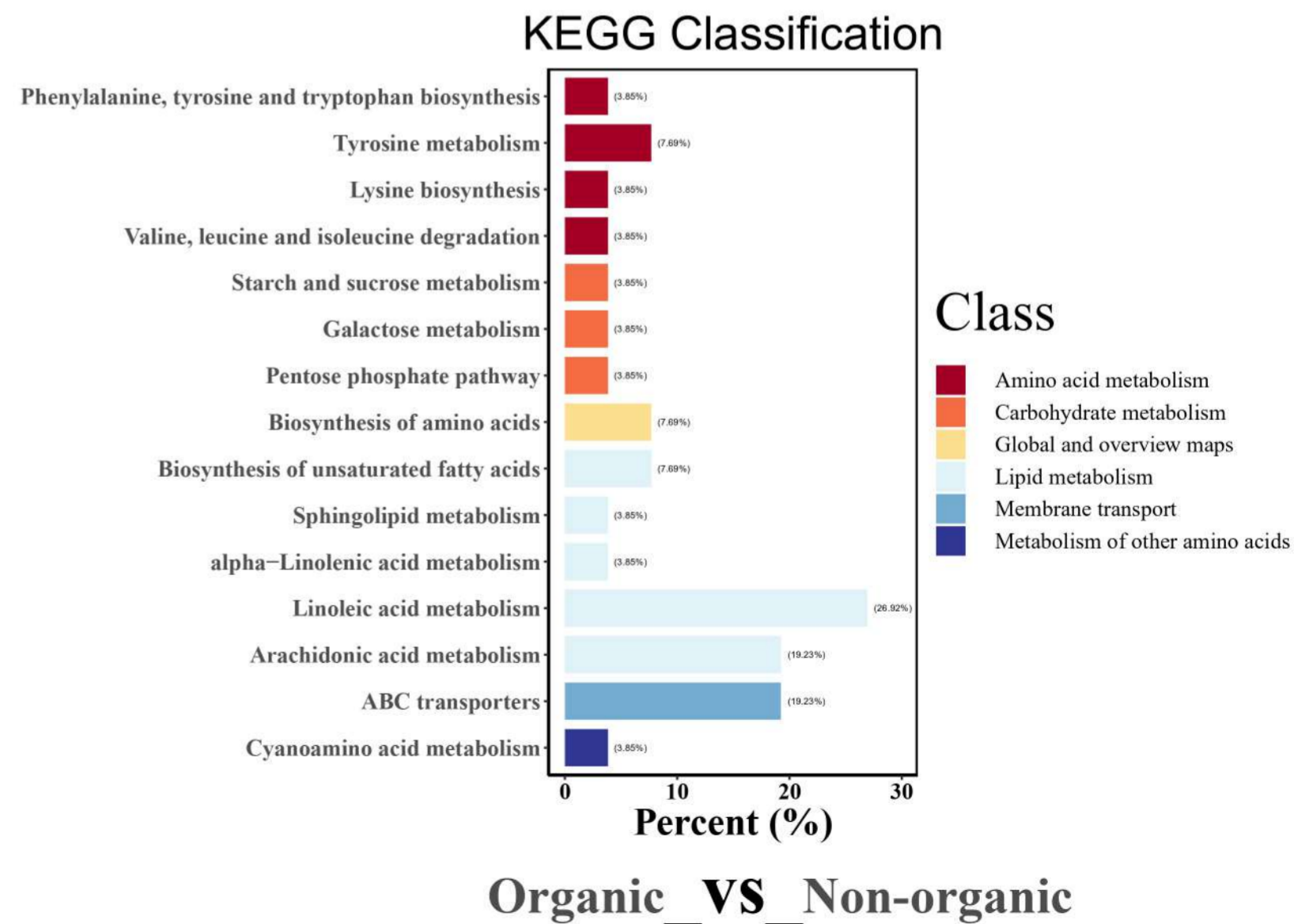

Supplement: Supplementary file 1 [file Data_Sheet_1.PDF]

A

● Fungi(F) ● Bacteria(B) — Positive — Negative

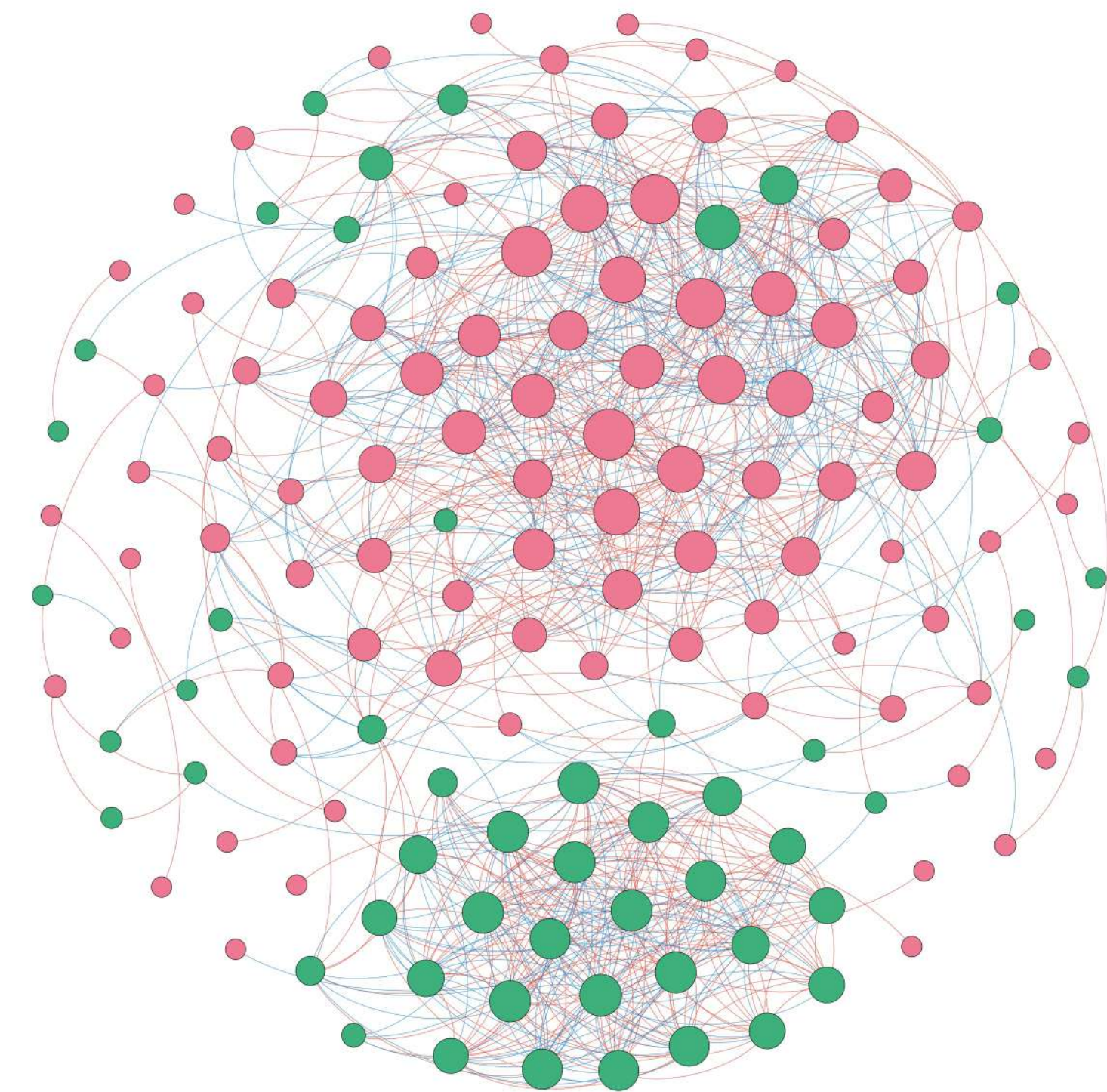

● F(94, 64.38%) Positive(590, 59.24%)  
 ● B(52, 35.62%) Negative(406, 40.76%)

B

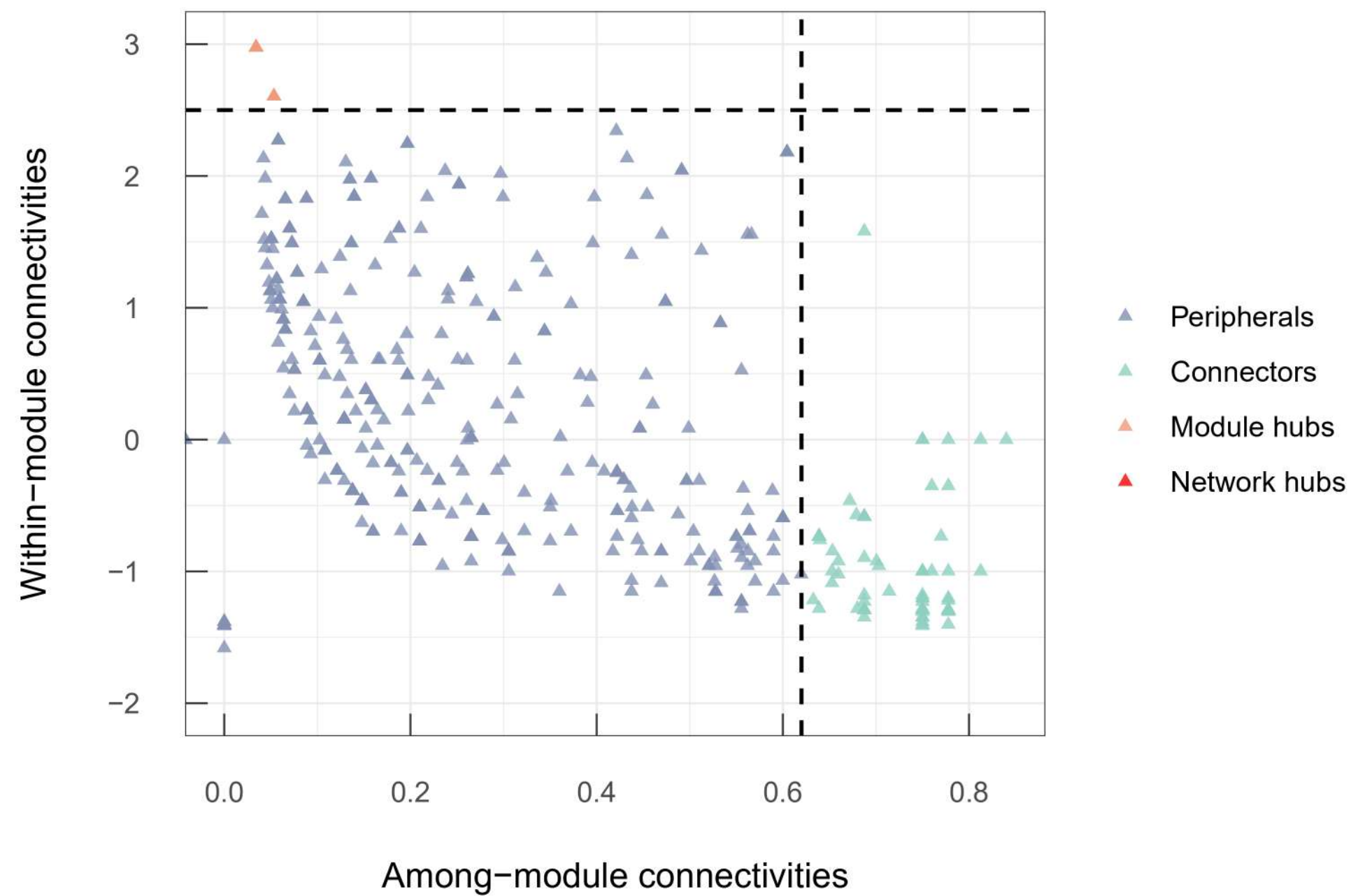

Supplement: Supplementary file 2 [file Data_Sheet_2.PDF]
